# Serodiscordant partnerships and opportunities for pre-exposure prophylaxis among partners of women and men living with HIV in St. Petersburg, Russia

**DOI:** 10.1371/journal.pone.0207402

**Published:** 2018-11-16

**Authors:** Natalia Gnatienko, Jennifer A. Wagman, Debbie M. Cheng, Angela R. Bazzi, Anita Raj, Elena Blokhina, Olga Toussova, Leah S. Forman, Dmitry Lioznov, Carly Bridden, Meg Sullivan, Kendall Bryant, Jeffrey H. Samet, Judith I. Tsui

**Affiliations:** 1 Department of Medicine, Section of General Internal Medicine, Clinical Addiction Research and Education (CARE) Unit, Boston Medical Center, Boston, MA, United States of America; 2 Department of Medicine, Division of Global Public Health, University of California San Diego School of Medicine, IOA Building, San Diego, CA, United States of America; 3 Department of Biostatistics, Boston University School of Public Health, Boston, MA, United States of America; 4 Department of Community Health Sciences, Boston University School of Public Health, Boston, MA, United States of America; 5 Laboratory of Clinical Pharmacology of Addictions, First Pavlov State Medical University, St. Petersburg, Russian Federation; 6 Biostatistics and Epidemiology Data Analytics Center, Boston University School of Public Health, Boston, MA, United States of America; 7 Smorodintsev Research Institute of Influenza, St. Petersburg, Russian Federation; 8 Department of Infectious Diseases and Epidemiology, First Pavlov State Medical University, St. Petersburg, Russian Federation; 9 Department of Medicine, Section of Infectious Diseases, Boston University School of Medicine/Boston Medical Center, Boston, MA, United States of America; 10 HIV/AIDS Research, National Institute on Alcohol Abuse and Alcoholism, National Institute of Health, Bethesda, MD, United States of America; 11 Department of Medicine, Section of General Internal Medicine, Clinical Addiction Research and Education Unit, Boston University School of Medicine/Boston Medical Center, Boston, MA, United States of America; 12 Department of Medicine, Section of General Internal Medicine, University of Washington School of Medicine and Harborview Hospital, Seattle, WA, United States of America; New York Blood Center, UNITED STATES

## Abstract

**Objective:**

To describe the frequency of being partnered and having an HIV-negative partner, and whether this differed by gender, among a cohort of persons living with HIV (PLWH) who have ever injected drugs; to describe awareness of HIV pre-exposure prophylaxis (PrEP) and perceived partner interest in PrEP.

**Setting:**

Secondary analyses of an observational cohort study of PLWH who have ever injected drugs in St. Petersburg, Russia.

**Methods:**

Primary outcomes were 1) being partnered and 2) being in a serodiscordant partnership. The main independent variable was gender. Multivariable GEE logistic regression models were fit for binary outcomes, adjusted for age, income, education, and recent opioid use. Descriptive analyses were performed for partners’ HIV status, substance use, sex risk behaviors, and awareness of PrEP for a subset of participants.

**Results:**

At baseline, 50% (147/296) reported being in a partnership, and of those, 35% were in a serodiscordant partnership. After adjustment, women had significantly higher odds of being partnered compared to men (aOR = 3.12; 95% CI: 1.77, 5.51), but there were no significant gender differences in the odds of being in a serodiscordant partnership (aOR = 0.58; 95% CI: 0.27, 1.24). Among a sub-sample of participants queried (n = 56), 25% were aware of PrEP for prevention of sexual HIV transmission and 14% for prevention of injection-related transmission.

**Conclusion:**

Although half of our sample were partnered and one third of these partnerships were serodiscordant, PrEP awareness was low. Substantial opportunities for HIV prevention exist among PLWH who have ever injected drugs in Russia and their HIV-negative partners. Given the high proportion of HIV-negative partners among this ART-naïve sample, efforts to address the associated inherent risks, such as couples-based interventions, are needed to increase condom use, PrEP awareness, or uptake of other HIV-prevention modalities (e.g., ART for the HIV-positive partner).

## Introduction

Incidence of HIV infection continues to rise in parts of the world where transmission is driven by injection drug use [1]. People who inject drugs (PWID) account for 30% of new HIV infections outside of sub-Saharan Africa [2]. Russia, with already one of the highest rates of HIV infection, is one of the few countries where HIV incidence is increasing [3]. Among the estimated 900,000–2,000,000 people living with HIV (PLWH) in Russia [4,5], up to 80% are PWID [6], and 47% of new HIV cases with a known mode of transmission are among PWID [4]. As such, interventions to slow HIV transmission among PWID in Russia are needed.

Antiretroviral pre-exposure prophylaxis (PrEP) with tenofovir disoproxil fumarate / emtricitabine (TDF/FTC) prevents HIV transmission within serodiscordant heterosexual couples [7–9] and HIV acquisition among at-risk PWID [10,11] and men who have sex with men (MSM) [12], and is currently recommended for HIV prevention in those populations [13]. Sexual partners of PLWH who have ever injected drugs are prime candidates for consideration of PrEP, as research demonstrates that both sexual and drug-related risk behaviors often occur simultaneously in such partnerships and create the potential for an injection drug driven epidemic to transition to the general population [14–16]. For countries like Russia where injection drug use is a primary driver of HIV transmission and linkage to antiretroviral therapy (ART) among PLWH who have ever injected drugs is suboptimal [17], offering PrEP to uninfected partners of these individuals could be an important strategy for limiting the spread of HIV, as it would help mitigate the transmission risks for uninfected partners that are associated with lack of viral suppression among PLWH [18,19]. Although PrEP is not yet available in Russia, evidence from rapid PrEP roll-out to MSM in New South Wales, Australia suggests that it could help reduce HIV incidence in other concentrated epidemic settings like Russia [20].

Female partners of male PLWH who have ever injected drugs may be at particularly high risk for HIV transmission because women may experience greater risk for HIV acquisition than men [21]. Furthermore, women may experience heightened HIV risk from injection drug use; research in the U.S. has shown that women who inject are more likely to report a regular sex partner who also injects compared to men [22], and having an intimate injection partnerships (i.e. sexual partnership with a partner who injects) confers increased likelihood of high risk injecting practices such as receptive syringe sharing [14]. In some studies of PWID, women have had higher hepatitis C virus (HCV) [22] and HIV incidence than men [23]. The frequency of partnerships, and partner’s HIV status, among women and men living with HIV who inject drugs has been relatively unexplored.

The primary aim of this exploratory study was to describe partnerships, and specifically serodiscordant partnerships, over time among a cohort of PLWH who have ever injected drugs and were ART-naïve at enrollment from St. Petersburg, Russia and assess differences between women and men. Based on existing literature, we hypothesized that, compared to male PLWH, female PLWH would have higher odds of being in a sexual partnership, but lower odds of being in a serodiscordant partnership as we assume a higher risk of having been infected through their partner. Secondary aims were to describe, among partnered participants, the frequency of condomless sexual episodes with partners, partners’ injection drug use status, and participants’ own PrEP awareness.

## Methods

### Study design and participants

We performed a secondary analysis of baseline and longitudinal follow-up data from the Russia ARCH cohort, a prospective observational study conducted in St. Petersburg. Russia ARCH is part of the Uganda, Russia, Boston Alcohol Network for Alcohol Research Collaboration on HIV/AIDS (URBAN ARCH) Consortium and was initially established to assess the association between alcohol consumption and biomarkers of inflammation, and also includes a nested randomized controlled trial of zinc supplementation, as previously described [24]. Participants were recruited between November 2012 and June 2015 from clinical HIV and addiction care sites, and non-clinical sites in St. Petersburg, Russia. Snowball recruitment was also utilized, where existing study participants referred their friends or acquaintances to be screened for the study. Eligibility criteria included the following: 18–70 years old; documented HIV-infection; documented ART-naïve at baseline; the ability to provide contact information for two contacts to assist with follow-up; stable address within 100 kilometers of St. Petersburg; and possession of home or mobile phone. Participants were excluded if they were not fluent in Russian or had a cognitive impairment resulting in the inability to provide informed consent. For the current study, the sample was restricted to Russia ARCH participants who acknowledged current and/or past injection of drugs defined as reporting history of injection drug use prior to their HIV diagnosis and/or injecting drugs in the past 30 days. For the descriptive sub-study on partners’ behaviors and PrEP awareness, the Russia ARCH sample was further restricted to participants who reported being in a partnership and agreed to answer supplemental questions during a study visit. The study was conducted in accordance with the Helsinki Declaration of 1975, as revised in 2000. The Institutional Review Boards of Boston University Medical Campus and First St. Petersburg Pavlov State Medical University approved this study and all participants provided written informed consent.

### Data collection

Participants were assessed at baseline, 12- and 24-months post enrollment. The baseline assessment included: demographics [25]; sex partners and behaviors [26]; alcohol 30-Day Timeline Follow Back [27]; drug use (modified Risk Behavior Survey) [28, 29]; and VR-12 Health Survey [30]. All assessments were conducted by trained research assessors and administered in Russian. Particularly sensitive sections of the assessment, including sex behaviors, were self-administered by the participant. Questions on partner-specific sexual behaviors and PrEP awareness were administered at one time-point in a subsample of participants who reported being partnered.

### Measures

#### Outcome Measures

The two primary outcomes of interest were self-report of: 1) being in a partnership, defined as being married, in a domestic partnership/living with partner or in a “long-term relationship,” the definition of which was left up to the discretion of the participant; and 2) having an HIV serodiscordant partnership, defined as current partner being HIV-negative as reported by the participant (versus partner is HIV-positive or unknown status).

Among the subgroup of participants who reported having a current partner and who answered supplementary questions, we also describe the following: 1) partners’ history of injection drug use, 2) specific sex risk behaviors with partners, 3) awareness of PrEP for preventing sexual and injection-related HIV transmission (“Have you heard of HIV-negative people taking HIV drugs [Pre-Exposure Prophylaxis or ‘PrEP’] to reduce their chances of getting HIV infection through having sex?”; “Have you heard of HIV-negative people taking HIV drugs [Pre-Exposure Prophylaxis or ‘PrEP’] to reduce their chance of becoming HIV infected as the result of injecting drugs?”), and 4) perception that their partner would be interested in PrEP (“If available, how willing do you think your partner would be in taking drugs to prevent her/himself from becoming HIV infected?”).

#### Main Independent Variable

The main independent variable of interest was female gender (the survey did not assess if individuals were transgender or non-binary).

#### Covariates/demographics

The following variables were included in analyses: age, education (up to a 9^th^ grade education or greater than 9^th^ grade), monthly income, income below the sample median (20,000 rubles/month; approximate equivalent US $345 as of 2017), current (past 30 day) opioid and/or heroin use, past month heavy drinking (National Institute on Alcohol Abuse and Alcoholism risky drinking criteria: > four standard drinks in a day [or > 14 standard drinks/week] for men and > three/day [or > seven/week] for women), CD4 cell count and HIV viral load results, past 90-day vaginal/anal/oral sex, past 90-day condomless sex, any report of same-sex sexual partnership, number of female and male sex partners on the past 6 months, and any transactional sex in the past 12 months (receiving or providing goods in return for sex).

### Statistical analysis

Descriptive statistics were used to characterize study participants overall and by gender at baseline, and chi-square and Student’s t tests were used to assess differences between groups. We tabulated the proportions of participants who reported being partnered (versus not partnered) and having an HIV serodiscordant partner (versus having a seroconcordant partner or partner whose status was unknown), overall and stratified by gender. We fit generalized estimating equations (GEE) logistic regression models to evaluate the association between gender and the outcomes, partnership and serodiscordant partnership, adjusting for age, education, income, past 30 day opioid and/or heroin use and time since baseline visit. Confirmatory analyses additionally controlled for receipt of intervention in the nested RCT. The models were fit using a logit link and standard errors are based on the empirical-sandwich estimator. All 296 participants meeting eligibility criteria were included in the primary analyses. All available outcomes were included with one exception (one participant missing baseline income was excluded from the adjusted model). Odds ratios (OR) and 95% confidence intervals (CI) are reported from the logistic regression models. Two-tailed tests and a significance level of 0.05 were used for all hypothesis testing. All analyses were conducted using SAS 9.3 [31].

For the subgroup of participants with a current partner, descriptive statistics characterized study participants and responses to supplemental questions, both overall and by gender. We tabulated the number and proportion of participants whose partners had ever injected drugs, who were aware of PrEP to prevent sexual and injection-related HIV transmission, and who believed their serodiscordant partner would be “very likely” to take PrEP. Means and standard deviations were calculated for the number of sexual encounters (vaginal or anal) in the past 90 days and the percentage of encounters for which a condom was used.

## Results

The sample was comprised of 296 PLWH who had ever injected drugs and were ART-naïve at baseline. At baseline, the sample had the following characteristics: mean age 33 years (range 20–50); 26% (77/296) female; 23% (67/296) ≤ 9th grade education; median income was 20,000 rubles (25^th^-75^th^ percentile: 5,000–30,000); median CD4 cell count 470 cells/uL (25^th^-75^th^ percentile: 304–702) (n = 203); and a median HIV viral load was 20306 copies/mL (25^th^-75^th^ percentile: 2856–113660) (n = 293). At baseline, 41% (121/296) reported opioid and/or heroin use in the past 30 days and 70% (207/296) reported heavy alcohol use as defined by NIAAA at-risk drinking amounts in the past 30 days. [32] Compared to men, women were significantly younger and more likely to report an income below the sample median; median CD4 cell count appeared higher for women, although not significantly different (Table 1).

**Table 1:** Baseline demographics and HIV factors in a cohort of PLWH not on ART who inject drugs in St. Petersburg, Russia (n = 296).

	Overall (n = 296)	Female (n = 77)	Male (n = 219)	p-value
Age, Mean (SD)	33.4 (4.8)	31.4 (4.2)	34.1 (4.8)	< .001
>9th grade education, n (%)	229 (77%)	56 (73%)	173 (79%)	0.26
Monthly income in rubles, Mean (SD)	20017 (19895)	13211 (13180)	22379 (21266)	<0.001
Income below median, n (%)	147 (50%)	52 (68%)	95 (44%)	<0.001
Past month opioid and/or heroin use n (%)	121 (41%)	35 (46%)	86 (39%)	0.34
Past month heavy drinking, n (%)	207 (70%)	59 (77%)	148 (68%)	0.14
CD4 cell count^a^, Median (25^th^-75^th^ percentile)	470.3 (304, 702)	523.5 (299, 698)	465.0 (305, 710)	0.25
HIV viral load^b^, Median (25^th^-75^th^ percentile)	20306 (2856, 113660)	11031 (1264, 90827)	22734 (3414, 117332)	0.07
Partnered, n (%)	147 (50%)	52 (68%)	95 (43%)	<0.001
Partner HIV status, n (%)				0.02
Positive	89 (61%)	35 (67%)	54 (57%)	
Negative	51 (35%)	12 (23%)	39 (41%)	
Unknown	7 (5%)	5 (10%)	2 (2%)	
Past 90 days number of times vaginal sex				0.002
N	292	76	216	
Mean (SD)	18.9 (30.2)	28.1 (41.4)	15.6 (24.4)	
Median (25th-75th percentile)	5.0 (1, 25)	9.5 (1, 44)	5.0 (0, 20)	
Past 90 days number of times anal sex				0.49
N	291	77	214	
Mean (SD)	1.0 (6.2)	0.6 (2.4)	1.2 (7.1)	
Median (25th-75th percentile)	0.0 (0, 0)	0.0 (0, 0)	0.0 (0, 0)	
Past 90 days number of times Oral sex				0.06
N	294	76	218	
Mean (SD)	8.5 (19.4)	12.1 (21.8)	7.3 (18.4)	
Median (25th-75th percentile)	1.0 (0, 10)	1.0 (0, 10)	0.0 (0, 7)	
Past 6 months number of female sex partners				< .001
N	293	77	216	
Mean (SD)	1.1 (1.6)	0.0 (0.1)	1.5 (1.7)	
Median (25th-75th percentile)	1.0 (0, 1)	0.0 (0, 0)	1.0 (1, 2)	
Past 6 months number of male sex partners				< .001
N	296	77	219	
Mean (SD)	0.6 (3.4)	2.1 (6.5)	0.0 (0.1)	
Median (25th-75th percentile)	0.0 (0, 0)	1.0 (1, 1)	0.0 (0, 0)	
Past 90 days any condomless sex, n (%)	139 (48.1%)	53 (70.7%)	86 (40.2%)	< .001
Any report of same-sex sexual partners, n (%)	4 (1.6%)	1 (1.3%)	3 (1.4%)	1.0
Transactional sex in past 12 months^c^, n (%)				<0.01
Gave money, drugs or alcohol in exchange for sex	30 (10.2%)	0 (0%)	30 (13.8%)	
Received money, drugs or alcohol in exchange for sex	4 (1.4%)	3 (3.6%)	1 (0.5%)	
Gave AND received money, drugs or alcohol in exchange for sex	6 (2.0%)	1 (1.3%)	5 (2.3%)	
None reported	254 (86.4%)	72 (94.7%)	182 (83.4%)	

^a^ n = 203 (female: n = 54, male: n = 149) due to missing laboratory data, as CD4 testing was added later in the study

^b^ n = 293 (female: n = 75, male: n = 218) due to missing laboratory data

^c^n = 294 (female: n = 76, male: n = 218) due to missing data

At baseline in the overall sample, 50% (147/296) reported being partnered, and of those, 35% (51/147) reported a serodiscordant partnership. Women were more likely to report having a partner than men (68% [52/77] v. 43% [95/21]), p = 0.0003), and were less likely to report having a partner who was HIV-negative (23% [12/52] v. 41% ([39/95], p = 0.02) (Table 1). Overall, the median number of vaginal sex episodes within the past 90 days was 5 (25^th^-75^th^ percentile: 1–25), and women reported nearly twice as many episodes of vaginal sex than men. Anal sex was almost never reported, irrespective of sex/gender or participant. Also, participants very seldom reported same-sex sexual partners, only 1.4% (n = 3/219) of men and only 1.3% of women (n = 1/77). In the overall sample 139/296 (48%) reported condomless sex in the past 90 days. The median number of sexual partners in the past 6 months reported for women and men was 1.

As shown in Table 2, we found that female gender was positively associated with being partnered in both the unadjusted model (OR = 2.97; 95% CI: 1.80, 4.90) and a model adjusted for age, education, income, past 30 day opioid and/or heroin use, and study visit (aOR = 3.12; 95% CI: 1.77, 5.51). Among the partnered participants (n = 180), being female was negatively associated with having a serodiscordant partner in the unadjusted analysis (OR = 0.43; 95% CI: 0.22, 0.84). After adjustment for age, income, education, past 30 day opioid and/or heroin use and visit, the association was attenuated and no longer significant (aOR = 0.58; 95% CI: 0.27, 1.24). Additional analyses controlling for whether the participant was randomized to receive zinc supplementation (via a nested intervention study) produced consistent results. The associations between being female and partnered were very similar after the additional adjustment for zinc (aOR = 3.12; 95% CI: 1.77–5.51), as was the association between being female and in a serodiscordant partnership (aOR = 0.57; 95% CI: 0.27–1.23).

**Table 2:** Unadjusted and adjusted relative odds for being partnered and being in an HIV-serodiscordant partnership over time in a cohort of PLWH who have ever injected drugs in St. Petersburg, Russia, GEE logistic regression model.

	Partnered (n = 296)				HIV- serodiscordant partnership (n = 180^a^)			
	Unadjusted OR (95% CI)	p-value	Adjusted OR (95% CI)	p-value	Unadjusted OR (95% CI)	p-value	Adjusted OR (95% CI)	p-value
Female	**2.97 (1.80, 4.90)**	**<0.001**	**3.12 (1.77, 5.51)**	**< .001**	**0.43 (0.22, 0.84)**	**0.013**	0.58 (0.27, 1.24)	0.16
Age			0.96 (0.92, 1.01)	0.14			1.04 (0.96, 1.11)	0.33
≤9th grade education			0.98 (0.56, 1.72)	0.96			0.76 (0.36, 1.63)	0.48
Income below median			0.57 (0.38, 0.85)	0.006			0.70 (0.39, 1.28)	0.25
Past 30 day opioid and/or heroin use			1.25 (0.80, 1.94)	0.33			0.55 (0.28, 1.06)	0.07
12 month visit			1.06 (0.80, 1.41)	0.69			0.72 (0.46, 1.13)	0.15
24 month visit			1.34 (0.95, 1.90)	0.10			1.17 (0.74, 1.86)	0.50

^a^ 180 reflects the subsample of individuals who reported at least 1 partnership during the study period.

Supplemental questions on number of condomless sexual encounters (vaginal and anal), use of injection drugs, and PrEP awareness were administered to a sub-sample of 56 participants from the main Russia ARCH study who had a follow-up visit remaining at the time the supplemental questions were added, and reported having a partner at that study visit. Table 3 provides characteristics of the sub-study (n = 56), which were similar to those of the overall sample (n = 296). For example, nearly a third of sub-study participants had been started on ART since their baseline visit, which was consistent with the overall cohort in which 30% initiated ART during the study. A similar proportion of sub-study participants also reported being in serodiscordant partnerships compared to the overall cohort at baseline (41% versus 35% respectively).

**Table 3:** Characteristics of a sub-sample of participants who reported being partnered (n = 56).

	Overall (n = 56)	Female (n = 21)	Male (n = 35)	p-value
Age, Mean (SD)	36 (6.5)	35.6 (8.7)	36.3 (4.9)	0.69
Years since HIV diagnosis, Mean (SD)	6.8 (5.1)	6.8 (5)	6.8 (5.2)	0.99
Partner’s status, n (%)				0.28
Positive	30 (54%)	14 (67%)	16 (46%)	
Negative	23 (41%)	6 (29%)	17 (49%)	
Unknown	3 (5%)	1 (5%)	2 (6%)	
Low income (0–25,000 RUB), n (%)	31 (55%)	17 (81%)	14 (40%)	<0.01
> 9^th^ grade education, n (%)	54 (96%)	20 (95%)	34 (97%)	1.0
CD4 cell count, Median (25^th^-75^th^ percentile)	359.3 (245, 563)	306.9 (207, 552)	429.4 (287, 574)	0.48
HIV viral load, Median (25^th^-75^th^ percentile)	13780 (250, 127961)	17158 (250, 135856)	7743 (250, 125643)	0.21
History of injection drug use or recent injection drug use, n (%)	45 (80%)	14 (67%)	31 (89%)	0.08
Past month injection drug use, n (%)	20 (36%)	9 (43%)	11 (31%)	0.39
Past month heavy drinking, n (%)	21 (38%)	8 (38%)	13 (37%)	0.94
Past 6 months ART use, n (%)	18 (32%)	8 (38%)	10 (29%)	0.46

Table 4 provides partner-specific HIV sex and drug risk behaviors and PrEP awareness for the sub-study. Nearly half (46%) reported that their partner injected drugs. The median number of sexual encounters in the past 90 days was 20 (25^th^– 75^th^ percentile: 5–30). The mean percentage of sexual encounters where a condom was used was relatively low (32%); there appeared to be significant differences by gender, with women reporting a lower proportion of sexual encounters with a condom. PrEP awareness was low: only 25% (14/56) had ever heard of PrEP for preventing sexual HIV transmission, and only 14% (8/56) had heard of it for preventing injection-related HIV transmission. There were no differences in PrEP awareness by gender. Interestingly, only a minority (10/26 or 39%) of participants with a serodiscordant (i.e. uninfected) partner thought their partner would be “very likely” to take PrEP.

**Table 4:** Partner-specific HIV risk behaviors and PrEP awareness among sub-sample of participants who reported being partnered (n = 56).

	Overall (n = 56)	Female (n = 21)	Male (n = 35)	p-value
Partner ever used injection drugs, n (%)	26 (46%)	12 (57%)	14 (40%)	0.39
Past 90 days vaginal or anal sex with current partner				0.39
N	53	19	34	
Mean (SD)	24.3 (23.1)	20.7 (20.2)	26.4 (24.6)	
Median (25th-75th percentile)	20 (5, 30)	20 (3, 40)	20 (10, 30)	
Percent of sexual encounters with partner where a condom was used, Mean (SD)	32% (45.4)	11% (28.9)	42% (48.3)	0.03
Awareness of PrEP to prevent HIV transmission through sex, n (%)	14 (25%)	5 (24%)	9 (26%)	0.87
Awareness of PrEP to prevent HIV transmission through injecting drugs, n (%)	8 (14%)	3 (14%)	5 (14%)	1.00
Serodiscordant partner would be “very likely” to take medication to prevent HIV infection, n (%)	10 (39%)	3 (43%)	7 (37%)	0.90

## Discussion

Among PLWH who have ever injected drugs in St. Petersburg, Russia, half were partnered at baseline (i.e. married, living together or in a stable long-term relationship), and approximately a third of those with partners reported having an HIV-negative partner, revealing substantial opportunities for HIV prevention with this population. As we hypothesized, a greater proportion of women were partnered than men, and among partnered participants, fewer women were in serodiscordant partnerships than men. After adjusting for age, income, education, and past month opioid and/or heroin use, being female was associated with three-fold greater odds of being partnered. In the subgroup of partnered participants, it appeared that participants’ uninfected partners could be at substantial risk for HIV acquisition through frequent condomless sex and injection drug use (i.e., approximately half of participants’ partners were also PWID). However, awareness of PrEP to prevent sexual and injection-related HIV transmission was low among both women and men in this sub-sample.

Our results highlight the substantial opportunities for HIV prevention among serodiscordant heterosexual partners of PLWH who have ever injected drugs in Russia. It is important to note that all HIV-positive participants in this cohort were ART-naive at baseline due to study eligibility criteria; thus, their HIV-negative sexual and injection partners at the time were at substantial risk for acquiring HIV. Detectable plasma HIV-1 RNA levels have clearly been shown to be a risk factor for transmission [18, 19]. Our results are also highly relevant when viewed in the context of recent research demonstrating that heterosexual HIV transmission is increasing in St. Petersburg [33]. HIV incidence rates among PWID in St. Petersburg are reportedly among the highest in the world [34], and high risk sexual behaviors are common among women and men in this population [35]. HIV-negative sexual partners of PLWH who have ever injected drugs are a potential “bridging population” allowing crossover of the epidemic to non-injection drug using populations [36, 37]. Thus there is a compelling public health argument to implement HIV prevention strategies for the partners of PLWH who have ever injected drugs in this setting, including: ART to achieve viral suppression in the infected partner, access to syringe service programs, opioid agonist therapy, enhanced education and condom distribution, and PrEP.

While the serodiscordant sexual partners of PLWH who have ever injected drugs are important candidates for PrEP due to the likelihood of overlapping injection-related and sexual HIV risk behaviors within these partnerships [14, 15], the feasibility of providing PrEP to HIV-negative partners of PWID in Russia and other parts of the world remains largely unknown due to limited research on PrEP for PWID in real-world settings. The results of this study suggest that there are major gaps in PrEP awareness and knowledge among PLWH in St. Petersburg. We are unaware of other studies on PrEP awareness in Russia; however, prior studies conducted in the U.S. and Canada have also demonstrated a low awareness of PrEP among PWID [38–41]. Specific concerns voiced by PWID regarding PrEP roll out include fear that PrEP implementation could detract from efforts to scale-up other evidence-based HIV prevention approaches such as access to sterile syringes and opioid agonist treatment [42]. Also, only a minority of participants in serodiscordant partnerships believed that their partners would be very interested in PrEP. While PrEP could be conceptualized as one component of the HIV prevention “toolkit” for the partners of PWID in Russia, more research is needed to understand the individual and community beliefs and circumstances that influence PrEP acceptability.

There are several limitations to this study. Our partnership definition (being married, in a domestic partnership/living with partner or in a long-term relationship) did not specify whether the partnership was sexual. While it is possible that some reported partnerships were not sexual in nature, most partnerships reported in the sub-study were sexual and unprotected sexual encounters within these partnerships were common. At the same time, participants may also have had multiple sexual relationships outside of primary partnerships, including non-heterosexual relationships, which were not captured. Information on the serostatus of participants’ partners was based on report and not confirmed with laboratory testing. In addition, some participants reported their partner’s status to be “unknown”, in which case we assumed the partner was also infected. However, this was an infrequent occurrence with only 5% of responses coded as such. Finally, our sample size for the supplemental questions on PrEP awareness was small; additional research is needed.

In summary, this study of PLWH who have ever injected drugs and were ART-naïve at baseline found that half were partnered (i.e., married, living with someone, or in a long-term relationship), and among partnered participants, approximately a third had an HIV-negative partner. Results also suggest that the partners of these PLWH who have ever injected drugs may be prime targets for PrEP, as they appear to be at high risk for acquiring HIV through unprotected sex and injection drug use. Yet awareness of PrEP, both for sexual and injection-related HIV prevention, was low among the HIV-positive partners in this sample. Aside from helping PLWH achieve viral suppression through ART, efforts are needed to increase access to a variety of HIV prevention methods—possibly including PrEP—for the at-risk, HIV-negative partners of PLWH who have ever injected drugs in St. Petersburg, Russia.

## Supporting information

S1 File Dataset. (CSV)
